# Supplementary material for: Left atrial reservoir strain is a marker of atrial fibrotic remodeling in patients undergoing cardiovascular surgery: Analysis of gene expression
Source: PLoS One. 2024 Jul 8;19(7):e0306323. doi: 10.1371/journal.pone.0306323 (PMC11230549; doi:10.1371/journal.pone.0306323)
Supplement: S1 Table — (DOCX) [file pone.0306323.s001.docx]

| Supplemental Table 1. Primers used in this study |
| --- |

| **_Symbol_** | **_Primer sequence_** |  | **_Product size_** | **_Accession no._** |
| --- | --- | --- | --- | --- |
| **_COL1A1_** | _F: CGTGACCTCAAGATGTGCCA_ | _R: CCGCCATACTCGAACTGGAA_ | _242bp_ | _NM_000088_ |
| **_COL3A1_** | _F: AGGGGAGCTGGCTACTTCTC_ | _R: TAGGAGCAGTTGGAGGCTGT_ | _267bp_ | _NM_000090_ |
| **_FN1_** | _F: ACCAACCTACGGATGACTCG_ | _R: TGCCACTGTTCTCCTACGTG_ | _331bp_ | _NM_212482_ |
| **_MMP2_** | _F: ATGACAGCTGCACCACTGAG_ | _R: ATTTGTTGCCCAGGAAAGTG_ | _174bp_ | _NM_004530_ |
| **_TIMP1_** | _F: TCTGGCATCCTGTTGTTGCT_ | _R: CGCTGGTATAAGGTGGTCTGG_ | _155bp_ | _NM_003254_ |
| **_TIMP2_** | _F: ATGCACATCACCCTCTGTGA_ | _R: CTCTGTGACCCAGTCCATCC_ | _177bp_ | _NM_003255_ |
| **_ACE_** | _F: TCTTCGCGCAGAGCTACAAC_ | _R: ATGTTGCTTAGCAGGGCGTT_ | _298bp_ | _NM_000789_ |
| **_TGFB1_** | _F: CTAATGGTGGAAACCCACAACG_ | _R: TATCGCCAGGAATTGTTGCTG_ | _209bp_ | _NM_000660_ |
| **_EDN1_** | _F: GCTCGTCCCTGATGGATAAA_ | _R: TTCCTGCTTGGCAAAAATTC_ | _216bp_ | _NM_001955_ |
| **_PDGFD_** | _F: GAACAGCTACCCCAGGAACC_ | _R: CTTGTGTCCACACCATCGTC_ | _193bp_ | _NM_025208_ |
| **_CTGF_** | _F: AGCAGCTGCAAGTACCAGTG_ | _R: CCAGGCAGTTGGCTCTAATC_ | _250bp_ | _NM_001901_ |
| **_NOX2_** | _F: TGGTGATGTTAGTGGGAGCA_ | _R: CTTTCCTGCATCTGGCTCTC_ | _196bp_ | _NM_000397_ |
| **_NOX4_** | _F: CTTCCGTTGGTTTGCAGATT_ | _R: TGAATTGGGTCCACAACAGA_ | _250bp_ | _NM_016931_ |
| **_IL1B_** | _F: GGGCCTCAAGGAAAAGAATC_ | _R: TTCTGCTTGAGAGGTGCTGA_ | _205bp_ | _NM_000576_ |
| **_TNF_** | _F: CCCGACTATCTCGACTTTGC_ | _R: AGGCCCCAGTTTGAATTCTT_ | _293bp_ | _NM_000594_ |
| **_NLRP3_** | _F: TCTGCTCATCACCACGAGAC_ | _R: GCAGCAAACTGGAAAGGAAG_ | _320bp_ | _NM_001079821_ |
| **_MCP-1_** | _F: CAGCCAGATGCAATCAATGCC_ | _R: TGGAATCCTGAACCCACTTCT_ | _190bp_ | _NM_002982_ |
| **_BAX_** | _Hs00180269_m1_ |  | _62bp_ | _NM_004324_ |
| **_NPPA_** | _F: GATTTCAAGAATTTGCTGGACCAT_ | _R: TTGCTTTTTAGGAGGGCAGATC_ | _227bp_ | _NM_006172_ |
| **_GAPDH_** | _F: CGAGATCCCTCCAAAATCAA_ | _R: TGTGGTCATGAGTCCTTCCA_ | _294bp_ | _NM_001289745_ |

COL1A1 (collagen type I alpha 1), COL3A1 (collagen type III alpha 1), FN1 (fibronectin 1), MMP2 (matrix metallopeptidase 2), TIMP1 (tissue inhibitor of metalloprotease 1), TIMP2 (tissue inhibitor of metalloprotease 2), ACE (angiotensin I converting enzyme), TGFB1(transforming growth factor-β1), EDN1 (endothelin-1), PDGFD (platelet derived growth factor D), CTGF (connective tissue growth factor), NOX2 (NADPH oxidase 2), NOX4 (NADPH oxidase 4), IL1B (interleukin 1 beta), TNF (tumor necrosis factor alpha), NLRP3 (NLR family pyrin domain containing 3), MCP-1 (monocyte chemoattractant protein 1), BAX (BCL2-associated X protein), NPPA (natriuretic peptide type A, atrial natriuretic peptide (ANP), GAPDH (glyceraldehyde-3-phosphate dehydrogenase)
