# Supplementary material for: Left atrial reservoir strain is a marker of atrial fibrotic remodeling in patients undergoing cardiovascular surgery: Analysis of gene expression
Source: PLoS One. 2024 Jul 8;19(7):e0306323. doi: 10.1371/journal.pone.0306323 (PMC11230549; doi:10.1371/journal.pone.0306323)
Supplement: S2 Table — (DOCX) [file pone.0306323.s002.docx]

**Supplemental Table 2**. Relationships between left atrial strain (LAS) and clinical data (age, sex, BMI) and echocardiographic findings in SR patients including PAF and total patients.

|  | **SR patients including PAF (n=84)** | |  |  | **All patients**  **(SR including PAF+AF）(n=118)** | |
| --- | --- | --- | --- | --- | --- | --- |
|  | **mLASr** | **mLAScd** | | **mLASct** | **mLASr** | **mLAScd** |
| **Age** | -0.214 (0.092) | **-0.427 (<0.001)***** | | 0.167 (0.188) | -0.115 (0.273) | **-0.352 (<0.001)***** |
| **Sex** | -0.123 (0.336) | **-0.348 (0.005)**** | | 0.071 (0.579) | -0.165 (0.113) | **-0.314 (0.002)**** |
| **BMI** | 0.245 (0.053) | 0.053 (0.679) | | 0.210 (0.095) | **0.247 (0.017)*** | 0.080 (0.441) |
| **LAD** | **-0.395 (0.001)***** | 0.001 (0.993) | | **-0.429 (<0.001)***** | **-0.537 (<0.001)***** | -0.085 (0.417) |
| **LVDd** | 0.005 (0.971) | **0.338 (0.006)**** | | **-0.251 (0.045)*** | -0.015 (0.887) | **0.310 (0.002)**** |
| **LVDs** | -0.133 (0.298) | 0.140 (0.269) | | **-0.253 (0.044)*** | -0.127 (0.227) | 0.137 (0.189) |
| **LVEF (bp)** | **0.367 (0.005)**** | 0.254 (0.055) | | 0.124 (0.355) | **0.312 (0.004)**** | **0.251 (0.020)*** |
| **E/e’** | -0.232 (0.069) | -0.169 (0.184) | | -0.057 (0.658) | **-0.216 (0.041)*** | **-0.298 (0.004)**** |
| **LAVI (bp)** | **-0.589 (<0.001)***** | -0.162 (0.246) | | **-0.585 (<0.001)***** | **-0.736 (<0.001)***** | **-0.236 (0.036)*** |
| **mLASr** | **-** | **0.543 (<0.001)***** | | **0.609 (<0.001)***** | **-** | **0.472 (<0.001)***** |
| **mLAScd** | **0.543 (<0.001)***** | - | | -0.180 (0.154) | **0.472 (<0.001)***** | - |
| **mLASct** | **0.609 (<0.001)***** | -0.180 (0.154) | | - | - | - |

*R*- and *P*-value are shown. **P*< 0.05, ***P*< 0.01, ****P*< 0.001
