# Supplementary material for: Left atrial reservoir strain is a marker of atrial fibrotic remodeling in patients undergoing cardiovascular surgery: Analysis of gene expression
Source: PLoS One. 2024 Jul 8;19(7):e0306323. doi: 10.1371/journal.pone.0306323 (PMC11230549; doi:10.1371/journal.pone.0306323)
Supplement: S3 Table — (DOCX) [file pone.0306323.s003.docx]

| **Supplemental Table 3.** Correlations of risk factors and drug use with LAS parameters |
| --- |

|  | mLASr  r value/*P* value | mLAScd  r value/*P* value | mLASct  r value/*P* value |
| --- | --- | --- | --- |
| Number of patients | 118 | 118 | 84 |
| Risk factor |  |  |  |
| Hypertension | -0.145/0.174 | -0.175/0.097 | -0.065/0.634 |
| Diabetes | 0.129/0.514 | -0.047/0.239 | 0.191/0.100 |
| Dyslipidemia | 0.068/0.527 | -0.137/0.195 | 0.146/0.273 |
| CKD | -0.053/0.620 | -0.064/0.545 | 0.011/0.856 |
| Drug use |  |  |  |
| β-blockers | -0.173/0.105 | -0.067/0.891 | -0.151/0.135 |
| Ca^2+^-blockers | 0.113/0.289 | 0.138/0.314 | 0.052/0.787 |
| ACE-I/ARB | -0.010/0.922 | 0.045/0.668 | -0.060/0.485 |
| Statins | 0.068/0.527 | -0.137/0.195 | 0.146/0.273 |
| Anti-diabetic drugs | 0.072/0.502 | 0.035/0.744 | 0.093/0.452 |
